# Utilisation de l'echographie doppler couleur dans la localisation de l'insertion du cordon ombilical et le devenir materno-foetal à la maternité de l'hôpital central de Yaoundé : une étude descriptive et analytique

**DOI:** 10.11604/pamj.2014.17.66.3473

**Published:** 2014-01-27

**Authors:** Jeanne Hortence Fouedjio, Florent Ymele Fouelifack, Maximilien Deutcho, Philip Nana Njotang, Robinson Mbu Enow, Robert John Ivo Leke

**Affiliations:** 1 Maternité Principale de l'Hôpital Central de Yaoundé, Cameroun; 2 Faculté de Médecine et des Sciences Biomédicales de l'Université de Yaoundé I, Cameroun; 3 Faculté de Médecine de L'université des Montagnes, Cameroun

**Keywords:** Echographie doppler couleur, sensibilité, spécificité, insertion du cordon ombilical, Color Doppler Ultrasound, sensitivity, specificity, umbilical cord insertion

## Abstract

**Introduction:**

L'échographie doppler couleur permet d'étudier l'insertion du cordon ombilical sur le placenta. Les études américaines et asiatiques montrent que les insertions anormales telles les insertions vélamenteuses et marginales sont associées à une élévation de la morbidité et de la mortalité périnatales. En Afrique et plus particulièrement au Cameroun, aucune étude n'a été publiée sur le sujet. D'où notre motivation à mener ce travail.

**Méthodes:**

Il s'agissait d'une étude descriptive et analytique qui s'est déroulée sur une période deux ans (2011-2012) à la maternité principale de l'Hôpital Central de Yaoundé. Cette étude a inclus 66 patientes qui ont subi chacune une échographie doppler couleur entre la 18^ième^ et 30^ième^ semaine de gestation, précisant le type d'insertion du cordon ombilical sur le placenta. A l'accouchement, un examen macroscopique du placenta a été réalisé afin de comparer le type d'insertion et réaliser les tests statistiques.

**Résultats:**

Des 66 grossesses étudiées, nous avons eu un pourcentage de visualisation du type d'insertion de 100%. Toutes les insertions étaient normales à l'échographie soit 20 centrales et 46 latérales. A l'examen macroscopique du placenta, nous avons obtenu 19 (28,8%) insertions centrales, 47(71,2%) insertions latérales ; aucune insertion anormale n'ayant été objectivée. Les tests statistiques nous permettent d'avoir une sensibilité de 95%, une spécificité de 97,8%, une exactitude de 98%, une valeur prédictive positive de 95% et une valeur prédictive négative de 97,8%. Pour ces insertions, nous n'avons pas retrouvé d'association entre le mode d'accouchement, le poids de naissance, et le Score d'apgar avec le type d'insertion du cordon ombilical.

**Conclusion:**

Nous avons conclu que l'échographie doppler couleur a une haute sensibilité et spécificité dans la détermination de l'insertion du cordon ombilical sur le placenta. Il n'y a pas d'association entre le type d'insertion et le devenir maternofoetal.

## Introduction

L'échographie obstétricale est une technique d'imagerie permettant d'identifier et de localiser le sac gestationnel, de déterminer le type de grossesse et la vitalité fœtale, de réaliser la biométrie, l'étude morphologique fœtale et placentaire [1, 2].

Les données épidémiologiques estiment l'incidence d'insertion vélamenteuse du cordon ombilical de 0,24-1,80% de toutes les grossesses, taux multiplié par 10 au cours des grossesses multiples. Des études japonaises révèlent l'incidence de l'insertion vélamenteuse du cordon de 0,73% au cours des grossesses monofoetales et de 6,3% au cours des grossesses multiples [3–7].

L'insertion vélamenteuse est associée à un risque majeur de prématurité, de retard de croissance intra-utérine, d'anomalie de rythme cardiaque fœtal et d'un faible score d'Apgar [5]. L'échographie Doppler couleur est importante pour la détermination de l'insertion du cordon ombilical et le diagnostic de la présence d'anomalies structurales du cordon [8–10].

Aux Etats Unis d'Amérique, une étude menée par l'American Roentgen Ray Society de Boston sur l'évaluation du site d'insertion du cordon ombilical sur le placenta grâce à l'échographie obstétricale a abouti sur une sensibilité globale de 69% et une spécificité de 100% dans la détermination de l'insertion du cordon ombilical, avec une exactitude de 91% pour la révélation des insertions anormales [11].

Les études en Afrique plus particulièrement au Cameroun ne sont pas documentées bien que la tendance semble être en faveur d'une grande sensibilité et spécificité de l'échographie doppler couleur à déterminer l'insertion du cordon ombilical sur le placenta ainsi que ses anomalies. Les données exactes sur la question restent inconnues dans notre pays.

Notre étude avait pour objectif principal de déterminer la sensibilité et la spécificité de l'échographie doppler à détecter l'insertion du cordon ombilical sur le placenta tout en évaluant le devenir materno-fœtal pour chaque type d'insertion.

## Méthodes

Il s'agissait d'une étude descriptive et analytique menée à la Maternité Principale de l'Hôpital Central de Yaoundé (HCY), pendant une période de deux ans (2011-2012 inclus) après l'obtention de la clairance d'éthique N°123/CNE/SE/2011. L'échantillonnage était consecutif. Après avoir obtenu le consentement éclairé signé de chaque participante, nous avons réalisé une échographie obstétricale et doppler couleur par voie abdominale entre la 18^ème^ et la 30^ème^ semaine de gestation pour déterminer la localisation du placenta et l'insertion du cordon ombilical. Ces examens échographiques ont été réalisés par un gynécologue obstétricien formé en techniques d'échographie Doppler obstétricale selon les normes américaines. L'échographe utilisé avait les caractéristiques suivantes : SONOSITE-M-TURBO -C60X / 5-3 Mhz, transducteur: Réf. C08189-75 *Made in USA*.

Après l'accouchement, nous avons noté les paramètres du nouveau-né, réalisé l'examen macroscopique du placenta précisant l'insertion du cordon ombilical, afin de la comparer aux données échographiques et effectuer les différents calculs. Les variables étudiées étaient : les caractéristiques socio-obstétricales, l'insertion du cordon sur le placenta à l'échographie et à l'examen du placenta, le mode d'accouchement, le poids des nouveau-nés, et le score d' APGAR.

La collecte des données s'est faite sur une fiche technique adaptée à l'étude. Les fiches d'enquête étaient saisies grâce au logiciel Microsoft Word. Pour intégrer les analyses, nous avons utilisé les logiciels SPSS version 20.0 et Epi Info version 7.1.2.0. Les differences étaient considérées comme significatives au seuil de 5% ( p<0,05 ).

## Résultats

Durant la période consacrée à notre étude, il a été effectué des échographies doppler couleur chez 71 gestantes, et nous avons pu assister à l'accouchement et à l'obtention des résultats complets de 66 accouchées.

La tranche d'âge de 20 à 24 ans était la plus représentée. La majorité des patientes était de religion chrétienne, 64/66 (97%). Dans cette population, la majorité des femmes étaient célibataires 43/66 (65,1%). La plupart étaient originaires de la région de l'ouest 37/66 (56%). Concernant le secteur d'activité, les ménagères étaient les plus représentées (Table 1).

**Table 1:** Caractéristiques sociales des 66 gestantes incluses dans notre étude

Caractéristiques	Effectifs n=66	Pourcentages
**Age maternel moyen**	26.37 (15-40)	**%**
[15-19]	5	7.6
[20-24]	22	33.3
[25-29]	21	31.8
[30-34]	16	24.2
[35-39]	1	1.5
≥40	1	1.5
**Religion**		
chrétienne	64	97
musulmane	2	3
Statut matrimonial		
Mariée	22	33.3
Célibataire	43	65.2
divorcée	1	1.5
**Origine ethnique**		
Centre	11	16.7
Est	3	4,5
littoral	2	3
Nord	3	4.5
Nord-ouest	3	4.5
Ouest	37	56.1
Sud	3	4.5
Sud-ouest	4	6,1
**Situation professionnelle**		
Sans profession	2	3.0
Secteur informel	10	15.2
Emploie rémunéré	12	18.2
Elève	3	4.5
Etudiante	15	22.7
Ménagère	23	34.8
cultivatrice	1	1.5

Dans la population d'étude, la parité moyenne est de 1,5 avec des extrêmes de 0 à 7; Les nullipares étaient les plus représentées. L'âge gestationnel moyen des parturientes était de 39 semaines, avec des extrêmes de 32 et 43 semaines. Cinq (7,6%) patientes ont accouché prématurément et 6 (9,1%) en post terme. Il n' y avait pas de différence statistiquement significative (Table 2).

**Table 2:** Caractéristiques obstétricales des 66 gestantes incluses dans notre étude

Caractéristiques	Effectifs : n=66			Pourcentages : %	
**Parité moyenne**	1.5 (0-7)				
nullipare	20			30.3	
Primipare	19			28.8	
Pauci pare	13			19.7	
Multipare	11			16.8	
Grande multipare	3			4.6	
**Terme (semaines)**	**Insertion du cordon**		**Risque relatif**	**Intervalle de confiance**	**P-value**
	**Centrale N=19(%)**	**Latérale N=47(%)**			
**<37**	1(5.3%)	4(8.5%)	0.6	0.06 – 5.18	0.651
**[37 - 42]**	15(78.9%)	40(85.1%)	0.9	0.71 – 1.2	0.543
**>42**	3(15.8%)	3(6.4%)	2.4	0.54 – 11.18	0.228

A l'échographie, nous avons identifié 69,7% (46/66) d'insertions latérales, suivie des insertions centrales 30,3%(20/66). Aucune insertion anormale (vélamenteuses ou marginale) n'a pu être identifiée. A l'examen macroscopique du placenta, aucune insertion anormale n'a été objectivée. Nous avons eu 71,2% (47/66) d'insertions latérales et 28,8% (19/66) d' insertions centrales. Nous avons observé une sensibilité de 95%, une spécificité de 97,8%, une exactitude de 98%, une valeur prédictive positive de 95% et une valeur prédictive négative de 97,5% (Table 3).

**Table 3:** Tableau comparatif des 66 insertions du cordon et tests statistiques

Insertion du cordon ombilical	A l’échographie : n=66(%)	Examen anatomopathologique n=66(%)	Sensibilité	Spécificité	Exactitude	VPP	VPN
Centrale	20 (30.3%)	19 (28.8%)	95%	97.8%	98%	95%	97.8%
Latérale	46 (69.7%)	47 (71.2%)					
Marginale	0 (0)	0 (0)					
Vélamenteuse	0 (0)	0 (0)					

VPP : Valeur prédictive positive ; VPN : Valeur prédictive négative

Trente-quatre placentas (soit 51,5%) étaient localisés sur la paroi postérieure contre 26 (soit 39,4%) localisations antérieures, et 4 placentas (soit 6,1%) étaient bas insérés. En ce qui concerne la localisation du placenta en fonction du type d'insertion du cordon ombilical sur le placenta, nous retrouvons 47,4% (9/19) d'insertions centrales et 36,2% (17/47) d'insertions latérales pour la localisation antérieure du placenta avec une différence statistiquement significative : (P=0,026). De même, pour la localisation postérieure du placenta, nous retrouvons 7/19(36,8%) d'insertion centrale et 27/47(57%) d'insertion latérale avec une différence statistiquement significative : (P=0,000) (Table 4).

**Table 4:** Localisation placentaire selon le site d'insertion du cordon omblical

Localisation du placenta	Insertion du cordon		Total (%)	Risque relatif	Intervalle de confiance	P-value
	Centrale N=19	Latérale N=47				
**Antérieur**	9(47.4%)	17(36.2%)	26(39.4%)	0,52	0.29 – 0.96	0.026
**Postérieur**	7(36.8%)	27(57.4%)	34(51.5%)	0,25	0.13 – 0.51	0.000
**Fundique**	1(5.3%)	1(2.1%)	2(3%)	1	0.14 – 7.9	1
**Bas inséré**	2(10.5%)	2(4.3%)	4(6.1%)	1	0.25 – 3.99	1

Cinquante-cinq femmes (83,3%) ont accouché par voie basse tandis que 11 (16,7%) ont accouché par césarienne. Le poids moyen était de 3259,55grs, avec des extrêmes compris entre 1520 grammes et 4700 grammes. Parmi les nouveau-nés, 6 (9,1%) ont eu un poids inférieur à 2500 grammes; le reste (60 soit 90,1%) ayant eu un poids supérieur à 3500 grammes. Soixante-quatre (97%) nouveaux-nés ont eu un score d'Apgar supérieur à 7 à la cinquième minute. Cependant deux nouveaux-nés ont eu un score d'Apgar nul (Table 5).

**Table 5:** Devenir maternofœtal des 66 gestantes incluses dans notre étude

Mode d'accouchement	Insertion du cordon ombilical à l'accouchement		Risque relatif	Intervalle de confiance	P-value
	Centrale n=19	Latérale n=47			
Voie Basse	15(27.3%)	40(72.7%)	0.92	0.71 – 1.20	0.543
c/s d'urgence	4(50%)	4(50%)	2.47	0.68 –8.89	0.158
c/s élective	0(0%)	3(100%)	0	-	0.260
**Apgar à la 5** ^**ième**^ **minute**					
0	0	2	-	-	0.361
[1 - 7]	0	0	-	-	-
>7	19	45	1.04	0.98 – 1.20	0.361
**Poids de naissance (grammes)**					
<2500	2(10.5%)	4(8.5%)	1.2	0.24 – 6.19	0.796
[2500-3500]	11(57.9%)	28(59.6)	0.9	0.61 – 1.5	0.900
>3500	6(31.6%)	15(31.9%)	0.9	0.45 – 2.1	0.979

c/s : Césarienne

## Discussion

L'âge moyen des femmes était de 26,4 ans avec des extrêmes de 15 et 40 ans. La tranche la plus représentée était celle de 20 à 24 ans. Notre population d 'étude est constituée majoritairement de chrétiennes (97%), des célibataires (65,1%), des femmes originaires de la région de l'Ouest (56%) et des ménagères (34,8%). Les nullipares étaient les plus représentées 20/66(30,3%), suivies par les primipares 19/66(28,8%) Le terme moyen à l'accouchement était de 39 semaines, avec des extrêmes de 32 et 43 semaines ; la majorité d'entre elles 57/66(86,4%), ayant accouché entre la 37^ème^ et la 42^ème^ semaine de gestation. Nous n'avons pas retrouvé une association entre le type d'insertion du cordon ombilical et la prématurité; probablement du fait que toutes nos insertions étaient normales. Toutes les échographies ont été réalisées entre la 18^ème^ et la 30^ème^ semaine de gestation. Nomiyama et al. [12] avaient effectué leur recherche entre la 18^ème^ et la 20^ème^ semaine ; Sepulveda et al. [13] ont mené leur étude entre la 16^ème^ et la 40^ème^ semaine ; Pretorius et al. [14] ont mené leurs travaux entre la 13^ème^ et la 40^ème^ semaine La majorité des placentas étaient de localisations postérieures34/66(51,5%), suivie des localisations antérieures 26/66(39,4%), et enfin les placentas bas insérés 4/66(6,1%).

Sepulveda et al. [13] retrouvaient 50%(417/832) de localisations postérieures, 48%(399/832) de localisations antérieures, 2%(16/832) respectivement de localisations latérales et basses. Ces résultats pouvant être justifiés par la grande taille de leur échantillon. Toutes les insertions du cordon ombilical ont été visualisées durant la réalisation de nos échographies 66/66(100%). Sepulveda et al. [13] retrouvaient une visualisation de 99%(825/832). Ceci pouvant s'expliquer par le fait que leurs échographies aient été réalisées jusqu'a la 40ème semaine de gestation.

Nomiyama et al. [12] ont eu une visualisation de 99,8%(586/587). Leurs échographies ayant été réalisées entre la 18^ème^ et la 20^ème^ semaine, cette différence pourrait s'expliquer par la taille de leur population. Nous avons retrouvé une différence statistiquement significative (P=0,026) entre la localisation antérieure du placenta et les insertions centrales et latérales ; de même qu'entre la localisation postérieure du placenta et les insertions centrales et latérales : (P=0,000). De plus, il existe respectivement un effet protecteur entre d'une part les localisations antérieures du placenta et les insertions centrales et latérales (RR=0,52 ; IC:0,29-0,96 ; P=0,026) et d'autre part les localisations postérieures et ces mêmes insertions (RR=0,25 ; IC : 0,13-0,51 ; P : 0,00).

La plupart des patientes ont accouché par voie basse 55/66(83,3%), le reste ayant accouché par césarienne. Les indications majeures de césarienne étaient les disproportions céphalopelviennes. Le poids moyen de naissance était de 3259,5grammes, avec des extrêmes de 1520 et 4700grammes. La tranche de poids de naissance la plus représentative était celle de 2500 à 3500grammes 39/66(59,1%). Le bébé ayant eu le seul petit poids de naissance de 1520 grammes a été issu d'une césarienne d'urgence à 32 semaines indiquée pour pré-éclampsie sévère.

La plupart (97%) des bébés ont eu un score d'Apgar supérieur à 7/10 à la 5ème minute. Cependant, nous avons noté deux cas de mortnés dont l'un issu d'une mère séropositive au VIH connue depuis 8 ans sans traitement, qui au cours de sa grossesse et même en intra partum a développé plusieurs épisodes de fièvre. L'autre a été issu d'une mère dont la grossesse a été mal suivie et qui a développé une fièvre intrapartale. Il n'existe pas de différence statistiquement significative entre le poids fœtal de naissance, l'Apgar et le mode d'accouchement en fonction du type d'insertion du cordon ombilical. Les tests effectués ont trouvé une sensibilité, spécificité et exactitude respectivement de 95%, 97,8% et 98% de l'échographie doppler couleur à déterminer l'insertion du cordon ombilical. Di Salvo et al. [11] pour une étude menée sur 360 grossesses retrouvent une sensibilité de 69%, une spécificité de 100% et une exactitude de 91% à déterminer l'insertion du cordon y compris les insertions vélamenteuses et marginales. Ceci pouvant s'expliquer par le fait que leurs échographies ont été réalisées entre 13 et 39 semaines de gestation. Or nous savons qu'à un âge gestationnel avancé, il nous devient difficile d'apprécier l'insertion du cordon du fait du fŒtus qui s'applique contre le placenta. Nomiyama et al. [12] sur une étude menée sur 587 grossesses en utilisant des échographes de marque Toshiba SSA 160 et 260 Tokyo Japan, trouvent une sensibilité de 100%, une spécificité de 99,8%, une valeur prédictive positive de 83%, une valeur prédictive négative de 100%. Ceci s'explique par le fait que leurs échographies ont été réalisées entre 18 et 20 semaines de gestation contrairement aux nôtres qui ont été effectuées jusqu'à 30 semaines.

Pretorius et al. [14] qui ont mené l'étude sur 917 grossesses en utilisant un échographe de marque Acuson Mountain View CA 128XP avec un transducteur 3,5-5 Mhz, visualisent l'insertion dans 96% des cas et trouvent une sensibilité de 46%, une spécificité de 95%, une valeur prédictive positive de 67% et une valeur prédictive négative de 17% de visualisation des insertions du cordon ombilical. Ces résultats s'expliquent par les faits que leurs échographies aient été réalisées d'une part précocement (13 semaines), et d'autre part tardivement (40 semaines). Ils établissent eux même que la visualisation de l'insertion est fortement influencée par l'âge gestationnel. Celle-ci allant de 67% entre 15 et 20 semaines à12% entre 36 et 40 semaines. Au cours de notre étude, nous n'avons pas objectivé d'insertions anormales (vélamenteuses et marginales), et même des vasa prævia. Ceci pouvant s'expliquer d'une part par le fait que leurs incidences dans la littérature sont faibles : 0,24% à 1,8% de toutes les grossesses pour les insertions marginales et vélamenteuses [3, 4] et 1/2000-3000 pour les vasa prævia [5, 6].

## Conclusion

L'échographie doppler couleur obstétricale aurait une sensibilité et une spécificité élevées dans la détermination de l'insertion du cordon ombilical. Il n'ya pas d'association entre le type d'insertion et le devenir maternofoetal.
